# Stigma towards mental health disorders - Has anything changed?

**DOI:** 10.1192/j.eurpsy.2024.264

**Published:** 2024-08-27

**Authors:** M. Subramaniam, S. Shahwan, E. Abdin, Y. B. Tan, S. Gunasekaran, B. Lim, B. Tan, S. Shafie, G. Schomerus, S. A. Chong

**Affiliations:** ^1^Research, Institute of Mental Health, Singapore, Singapore; ^2^University of Leipzig Medical Center, Leipzig, Germany

## Abstract

**Introduction:**

Stigma towards mental disorders has been shown to be a major obstacle to recovery and quality of life among people with psychiatric disorders. Despite significant advances in the treatment of mental disorders, stigma remains concerning to patients, caregivers, and healthcare professionals. Singapore is a city state in South-East Asia with a multi-ethnic population. A nation-wide campaign launched in 2018, Beyond the Label, focusing on addressing stigma and promoting social inclusion for persons with mental health conditions.

**Objectives:**

The aims of the current study were to (i) establish the dimensions of stigma and examine its correlates in the general population of Singapore using a vignette approach, and (ii) examine whether there was any change in stigma levels from 2016 to 2023.

**Methods:**

Data for the current study comes from an ongoing nation-wide, cross-sectional study of mental health literacy conducted in Singapore since September 2022. The study population comprises Singapore Residents aged 18–65 years who are currently living in Singapore. Respondents were randomly assigned and presented a vignette describing one of seven specific disorders: alcohol abuse, dementia, depression, depression with suicidality, gambling disorder, obsessive-compulsive disorder, and schizophrenia. Stigma was assessed using Personal and Perceived scales of the Depression Stigma Scale (DSS) (Griffiths et al. Br J Psychiatry; 2004 185 342-349), and the Social Distance scale (SDS) (Link et al. Am J Public Health 1999; 89 1328–1333).

**Results:**

2500 respondents who completed the survey were included in the current analysis. The mean age of the respondents was 42.8 years. A three-factor model comprising ‘weak-not-sick’, ‘dangerous/undesirable’, and ‘social distance’ provided acceptable fit. Multivariable linear regression analyses revealed that younger age, female gender, students, and dementia vignette were significantly associated with lower weak-not-sick scores while Malay and Indian ethnicity, lower education, and alcohol abuse and gambling disorder vignette were significantly associated with higher weak-not-sick scores. Those of Malay and Indian ethnicities and those with a family member or close friend who had problems similar to the person in the vignette were significantly associated with lower social distance scores.

A significant decrease (p<0.001) in all three factor scores was observed from 2016 to 2023 (Table 1).

**Table 1.** Mean stigma scores over time

	2016	2023
Factors	Mean (SD)	Mean (SD)
Weak-not-sick	10.2 (2.1)	9.5 (2.3)
Dangerous/undesirable	11.6 (2.8)	11.2 (2.7)
Social distance	12.0 (3.1)	11.6 (3.0)

**Conclusions:**

Our study found a significant decrease in stigma from 2016 to 2023 in the Singapore population which indicates the positive impact of anti-stigma initiatives in Singapore. Interventions must be co-developed with males, older adults, and those with a lower education to further reduce stigma in this multi-ethnic population.

**Disclosure of Interest:**

None Declared

